# Effect of Proparacaine 0.375%-Sodium Fluorescein 0.25% Eye Drop Mixture and Fluorescein Strip on Anterior Segment Parameters

**DOI:** 10.1155/2018/5926508

**Published:** 2018-09-05

**Authors:** Mustafa Doğan, Mehmet Cem Sabaner, Mehmet Akif Erol

**Affiliations:** Faculty of Medicine, Department of Ophthalmology, Afyon Kocatepe University, Afyonkarahisar, Turkey

## Abstract

**Purpose:**

To determine the effect of proparacaine 0.375%-sodium fluorescein 0.25% eye drop mixture and fluorescein strip on anterior segment parameters commonly used in routine ophthalmology practice.

**Methods:**

115 healthy volunteers without any systemic or ocular disease were divided into two groups. 57 volunteers were in the proparacaine 0.375%-sodium fluorescein 0.25% eye drop mixture group, and 58 volunteers were in the fluorescein strip group. Measurements (CCT (central corneal thickness), topographic pupil diameter, AD (aqueous depth), ACV (anterior chamber volume), ICA (iridocorneal angle), LLD (limbus-limbus distance), and CV (corneal volume)) were taken before and at 1, 5, 15, and 30 minutes after application.

**Results:**

59 (51.3%) participants were female, and 56 (48.7%) were male. The mean age of the drop application group was 26.88 ± 8.03, and the mean age of the strip application group was 26.33 ± 7.28. The mean CCT was 556 ± 32 *μ*m before drop application and 569 ± 30 *μ*m in the 1st minute, 560 ± 32 *μ*m in the 5th minute, and 559 ± 31 *μ*m in the 15th minute. The change was statistically significant (*p* < 0.001, *p*=0.005, and *p*=0.013, resp.). Before the strip application, mean CCT was 552 ± 36 *μ*m, while it increased to 556 ± 36 *μ*m at the 1st minute, and this change was statistically significant (*p*=0.002). The mean CV before dropping was 59.29 ± 3.53 mm^3^ and 60.62 ± 3.53 mm^3^ at the 1st minute and 59.66 ± 3.70 mm^3^ at the 5th minute, which was statistically significant (*p* < 0.001 and *p*=0.034). Mean topographic pupil diameters at the 1st, 5th, 15th, and 30th minute after application of drops and strips were not significantly changed compared to the preapplication values in the AD, ACV, ICA, and LLD measurements.

**Conclusions:**

Proparacaine 0.375%-sodium fluorescein 0.25% eye drop mixture and fluorescein strip application lead to a temporary change in CCT and CV of the anterior segment parameters. Anterior segment measurements with the Scheimpflug camera have to be done before topical fluorescein application.

## 1. Introduction

Correct measurement of anterior segment parameters such as central corneal thickness (CCT), iridocorneal angle (ICA), anterior chamber volume (ACV), and limbus-limbus distance (LLD) is crucial in some of anterior segment surgeries (refractive surgeries) and clinical follow-up of many anterior segment-related diseases (i.e., congenital or acquired corneal diseases and glaucoma follow-up). To obtain anterior segment measurements, currently, ultrasonic biomicroscopy, ultrasonic pachymetry, Scheimpflug–Placido disk imaging, interferometry, optical reflectometry, confocal microscopy, slit topography, and anterior segment optical coherence tomography systems are used [1–5].

The Sirius topography device (Costruzione Strumenti Ophthalmics, Florence, Italy) is used in combination with a Scheimpflug camera and Placido disk technology in evaluating anterior segment parameters (6). With this technology, it is possible to evaluate CCT, ICA, ACV, LLD, aqueous depth (AD), keratometry, pupillography, wavefront analysis, and topography of the anterior-posterior surface of the cornea [1, 6].

Fluorescein, 376.67 dalton weight, is a weak dibasic acid substance with a sodium salt content [7–10]. In routine ophthalmology practice, the fundus is used for fluorescein angiography, applanation tonometry, epithelial defect staining, and tear breakage time [9–11].

**Figure 1:**
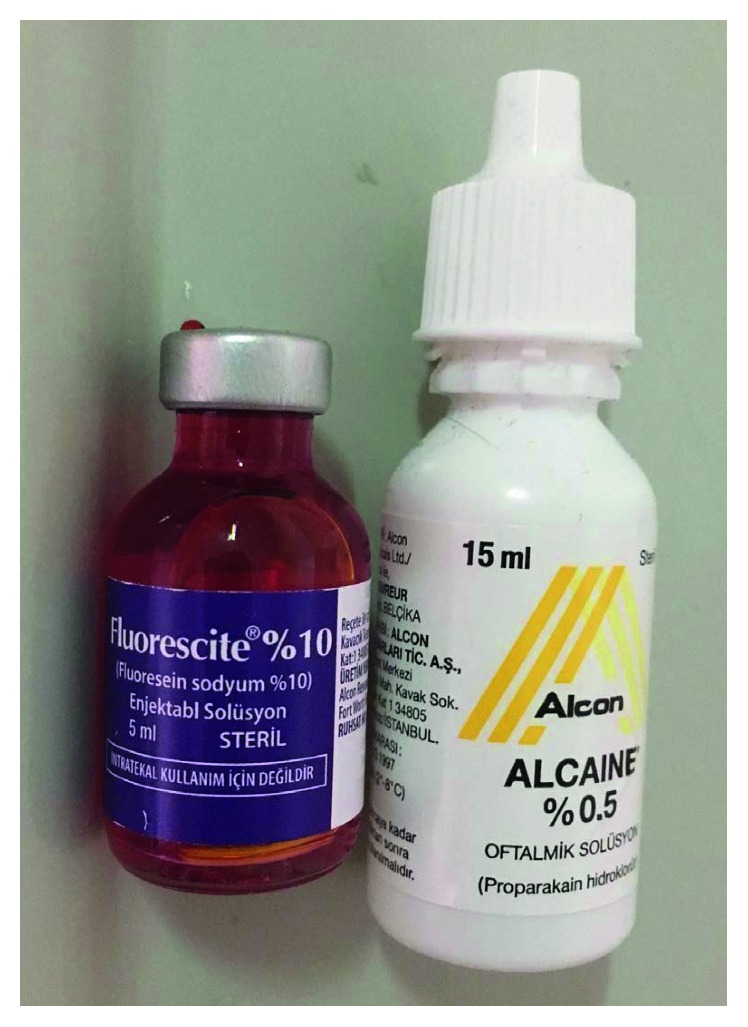
Proparacaine 0.375%-sodium fluorescein 0.25% eye drop mixture.

**Figure 2:**
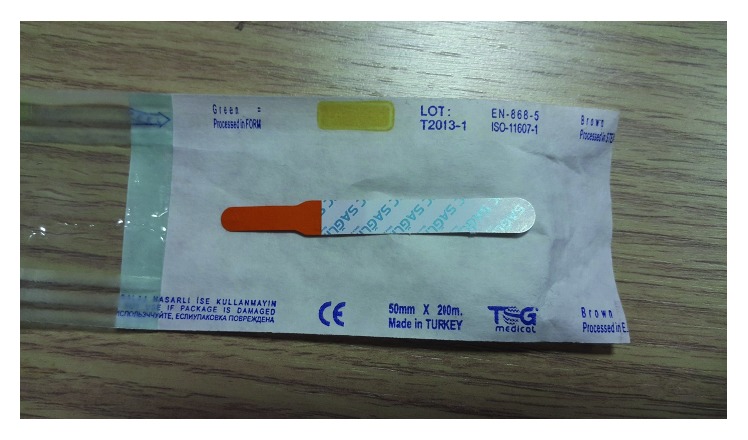
Fluorescein strip.

In this study, we aimed at determining the effect of fluorescein (frequently used as 0.375%-sodium fluorescein 0.25% eye drop mixture (Figure 1) and as a fluorescein strip (Figure 2) on corneal and anterior segment parameters.

## 2. Materials and Methods

Under the tenets of the Helsinki Declaration, written informed consent was obtained from all patients. Afyon Kocatepe University Ethical Committee approved the study.

This cross-sectional study included the right eyes of 115 volunteers. Whole patients received a full ophthalmologic examination. The inclusion criteria for all the participants consisted of spherical refraction between +1.0 and +1.0 diopters, best-corrected visual acuity of 20/20 or better, axial length (AL) of 525 mm, and normal intraocular pressure. The following exclusion criteria were applied: the presence of any ocular or systemic disease, having undergone any ocular surgery, a history of alcohol intake or smoking, and a history of taking any medication within the last 3 months because of the eye or systemic reasons.

### 2.1. Method

**Figure 3:**
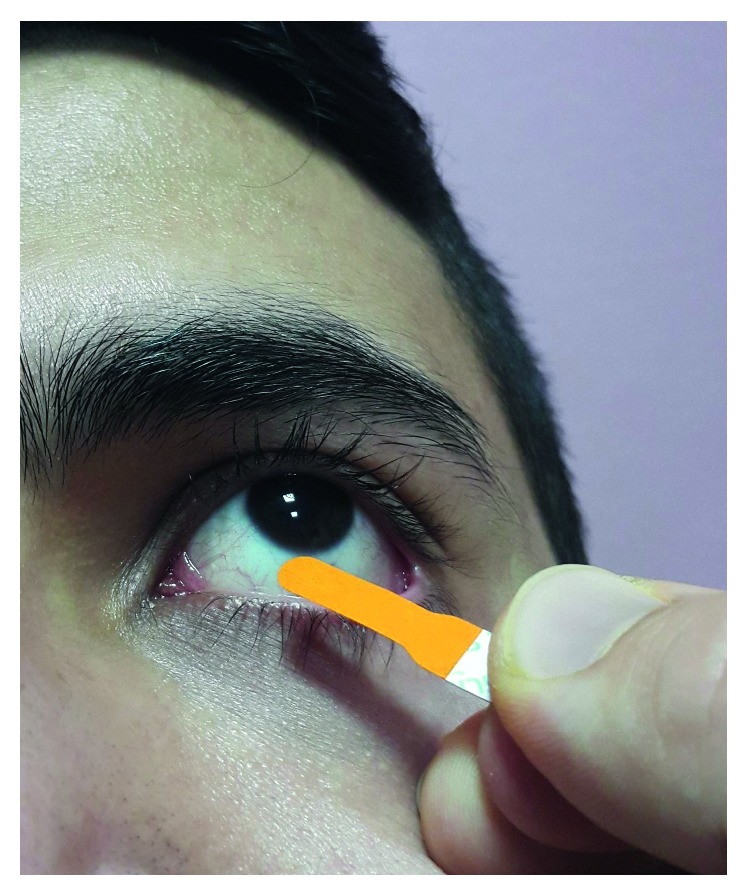
Fluorescein strip application.

Of the 115 volunteers, 57 were in with the proparacaine 0.375%-sodium fluorescein 0.25% eye drop mixture group and 58 were in with the fluorescein strip group (Figure 3). In the drop mixture group, 1 drop of the fluorescein drop mixture was applied to each eye. The eyes were kept closed for 1 minute after the drop was applied; after 1 minute, the eye was cleaned with a clean napkin; and measurements were taken. In the stripe group, the strip was left in the lower conjunctival fornix for 1 minute, then the eye was wiped with a clean napkin, and measurements were taken. By the same researcher, drop and strip group's measurements were taken before and the 1st, 5th, 15th, and 30th minutes after application. To avoid daily variation in corneal thickness, all measurements were taken at least 2 hours after awakening from sleep, from 09:30 to 11:30. The right eye of each patient was used for statistical analysis.

### 2.2. Sirius Scheimpflug–Placido Topography Device

Sirius topography device (Costruzione Strumenti Ophthalmologist, Florence, Italy) is a monochromatic 360-degree rotating Scheimpflug camera and a Placido disc combined with a 25-radial cross-sectional anterior segment analysis system (1,11). It provides tangential and axial curvature information of the anterior and posterior surface of the cornea, provides the global refractive power of the cornea, and provides pachymetry mapping and wavefront analysis of the cornea. The anterior and posterior surface of the cornea is scanned with UV-free 475 nm blue LED light and is followed by a pachymetric map (1,3). The Sirius system and the patient's pupil were aligned with the central fixation light along the visual axis. The patients were asked to lean back after each measurement, and the device was realigned to the cornea after every measurement. The patient was instructed to break once before each measurement, two consecutive measurements were taken, and the averages were taken to reduce the risk of the error due to measurement.

The mean CCT, topographic pupil diameter, AD, ACV, ICA, LLD, and CV (corneal volume) values measured at the 1st, 5th, 15th, and 30th minutes were compared with those before the application.

### 2.3. Statistical Analysis

The data obtained were recorded using the statistical package program (SPSS, version 22.0, SPSS, Chicago, USA). Descriptive statistical methods (mean and standard deviation) were used in the evaluation of the data. Comparisons of data were made using a paired *t*-test. The evaluations were made at the 95% confidence interval, and the *p* value less than 0.05 was considered a statistically significant difference.

## 3. Results

Of the 115 volunteers (115 eyes) included in the study, 59 (51.3%) were female and 56 (48.7%) were male. The mean age of the drop application group was 26.88 ± 8.03, and the mean age of the strip application group was 26.33 ± 7.28 (*p* > 0.05). There was no statistical difference between the two groups in terms of age, sex, and refraction (*p* > 0.05). The mean CCT before dripping was 556.04 ± 32.31 *μ*m and 569.18 ± 30.79 *μ*m at the 1st minute, 560.21 ± 32.23 *μ*m at the 5th minute, and 559.03 ± 31.96 *μ*m at the 15th minute, which was statistically significant (*p* < 0.001, *p*=0.005, and *p*=0.013, resp.) (Table 1). The mean CCT before stripping was 552.83 ± 36.51 *μ*m; at the 1st minute, it was 556.68 ± 36.73 *μ*m, and this change was statistically significant (*p*=0.002). The mean preanalytic CV was 59.29 ± 3.53 mm^3^ and 60.62 ± 3.53 mm^3^ at the 1st minute and 59.66 ± 3.70 mm^3^ at the 5th minute, which was statistically significant (*p* < 0.001 and *p*=0.034, resp.). The mean topographic pupil diameters, AD, ACV, ICA, and LLD measurements at the 1st, 5th, 15th, and 30th minutes after application of drops and strips were not significantly different from those before application.

## 4. Discussion

Goldmann applanation tonometry (GAT) in the diagnosis of glaucoma is important when the intraocular pressure is determined accurately and reliably by contact. In this system, which is mounted with biomicroscopy, topical anesthetics and fluorescein are applied in the patient's eye before measurement. And then measurement is performed using a 3.06 mm diameter corneal ring under the cobalt blue. The principle of determining the intraocular pressure is bringing two rings into the tip with the lever in the system [12, 13]. CCT is the second most important factor in the calculation of intraocular pressure, but when used with GAT, CCT is taken as a reference value at about 520 *μ*m. Since CCT is not the same in every individual, various formulations are used for correction of this condition, since measurements made with GAT will be lower in thin corneas and higher in thick corneas [14, 15]. In addition, accurate measurement and recognition of CCT and other anterior segment parameters (pupil diameter, AD, ACV, ICA, and LLD) are required in almost all anterior segment surgeons, especially refractive surgery except glaucoma diagnosis. On the contrary, topical fluorescein is administered before the test in determining tear breakage time, one of the tests for the diagnosis of dysfunctional tear syndrome [16]. Good anterior tear film stability is required before anterior segment surgery, and some surgeries (e.g., refractive surgery) are not performed if this is not the case.

On the contrary, there is information about the importance of control of the volume of fluorescein instillation; the amount of fluorescein in low concentrations does not provide corneal staining. But high concentrations of fluorescein increase corneal swelling and cause more rapid tear breakup and an iatrogenic “pseudostaining.” Therefore, fluorescein mixture drops are widely used at a concentration of 0.25%.

To measure anterior segment parameters with the Scheimpflug method, there are some articles which evaluated the effect of fluorescein strip and anesthetic substance + fluorescein mixture. Hirnschall et al reported an increase of 46.5 *μ*m on average in the first minute after applanation tonometry using proxymetacaine hydrochloride 0.5%-sodium fluorescein 0.25% before the applanation tonometer. However, they showed that the CCT returned to normal after an average of about 40 minutes [17]. Briggs et al. reported that when they applied a group fluorescein 2% and another group saline drop, the mean increase of 37.0 *μ*m in the CCT at the 1st minute in the fluorescein-infused group returned to normal at 60 minutes and reported no change in CCT in the saline drop-administered group [18]. In addition, some eyes in the fluorescein group showed a faster return to normal CCT values after 1 minute of saline wash. Parallel to these, in our study, we found an average increase of 13.1 *μ*m in the CCT at 1 minute, and we found that it returned to normal after 30 minutes. CCT and CV are used for corneal ectatic disease detection, before refractive surgery, and evaluation of the corneal status and corneal endothelial function [19–21]. Postoperative elevations of CCT and CV were observed in patients with long phacoemulsification, high power, and complicated cataract surgery, especially in patients with pseudoexfoliation syndrome, and the risk of corneal decompensation was high in these patients [22–24]. It should not be forgotten that an increase in CCT and CV due to topical fluorosis for any postoperative reason may cause suspicion of endothelial dysfunction and corneal edema, which may lead to the use of secondary drugs.

Mukhopadhyay et al. reported a changing ultrasound pachymetry after using proparacaine 0.50%-sodium fluorescein 0.25% mix, and they suggested that clinicians should avoid contact procedures before obtaining topographic maps of corneal thickness using scanning-slit and Scheimpflug devices [25]. In the same way, we have also detected pachymetric changes in the drop mix group, and we confirm the same recommendation.

The CCT and CV changes we detect in our study may be attributed to the intense presence of fluorescence in the tear film that the Scheimpflug imaging system perceives as the cornea rather than the true anatomic corneal changes. We think that the UV-free 475 nm blue LED light used in the measurement of the topography device causes interference in these parameters due to the yellow-green fluorescence caused by the fluorescence of the ocular surface. In addition, Zhuang et al., in patients with dysfunctional tear syndrome and healthy volunteers, applied 0.1% drop of fluorescein and measured CCT with Pentacam (Oculus, Dudenhofen, Germany) and found that the premeasurement difference was lower in patients with dysfunctional tear syndrome and attributed it completely to the tear film layer [26]. However, they evaluated the effect of fluorescence caused by blue light and fluorescein interaction as tear film thickness. It is known that the tear film layer becomes more visible after the application of the fluorescein drops, and thus, the information about the tear film can be obtained indirectly [16, 26–28].

On the contrary, we have found a temporary change in central corneal thickness in the eye drop mixture group (the 1st, 5th, and 15th minute after application) and corneal volume (the 1st and 5th minute after application). However, in the strip group, only changes in central corneal thickness appeared 1 minute after application. We think that the difference was due to the proparacaine from the drop mixture group. While only the fluorescein effect was seen in the strip group, in the drop mixture group, there was also the swelling volume effect of proparacaine together with fluorescein.

Limitations of our work are as follows: the effects of the fluorescein in the other anterior segment measurement techniques cannot be predicted because the results and measurements in the diseases that concern the anterior segment are made in one device only because they are made in healthy volunteers. In future studies, the effect of fluorescein can be investigated by comparing different topical anesthetic blended components with different disease groups and different devices.

In conclusion, proparacaine 0.375%-sodium fluorescein 0.25% eye drop mixture and fluorescein strip application caused approximately 10–15 microns and 1 mm^3^ but significant amount of corneal swelling in CCT and CV. Therefore, clinicians should avoid using fluorescein drop mixture or strip before obtaining topographic maps of corneal thickness or corneal volume using the Scheimpflug device because of the effect on CCT and CV may be greater than 10 *μ*m in cases.

## Figures and Tables

**Table 1:** Comparison of anterior segment parameter measurements in eye drop and strip groups.

	Before application	1st minute	5th minute	15th minute	30th minute	*p* ^*∗*^ value			
						*p*1	*p*2	*p*3	*p*4
*Eye drop group (n: 57; 29 female, 28 male) (mean *±* standard deviation)*									
CCT (*µ*m)	556.04 ± 32.31	569.18 ± 30.79	560.21 ± 32.23	559.03 ± 31.96	557.98 ± 32.66	*p * **<0** **.001**	**0**.**005**	**0**.**013**	0.100
Topographic pupil diameter (mm)	3.43 ± 0.75	3.42 ± 0.61	3.42 ± 0.59	3.42 ± 0.60	3.42 ± 0.65	0.603	0.598	0.599	0.664
AD (mm)	3.01 ± 0.31	3.02 ± 0.30	3.02 ± 0.30	3.02 ± 0.30	3.02 ± 0.30	0.351	0.446	0.458	0.061
ACV (mm^3^)	150.37 ± 28	151.73 ± 28	151.32 ± 29	151.50 ± 27	149.96 ± 34	0.073	0.281	0.301	0.838
ICA (angle)	40.49 ± 6.25	40.47 ± 6.11	40.34 ± 5.9	40.31 ± 6.0	40.55 ± 6.34	0.946	0.665	0.836	0.810
LLD (mm)	12.22 ± 0.65	12.17 ± 0.53	12.27 ± 0.72	12.20 ± 0.57	12.21 ± 0.65	0.403	0.572	0.830	0.925
CV (mm^3^)	59.29 ± 3.53	60.62 ± 3.53	59.66 ± 3.70	59.30 ± 3.62	59.39 ± 3.94	*p * **<0** **.001**	**0.034**	0.283	0.589

*Strip group (n: 58; 29 female, 29 male) (mean ± standard deviation)*									
CCT (*µ*m)	552.83 ± 36.51	556.68 ± 36.73	554.01 ± 37.42	553.85 ± 35.97	552.38 ± 35.09	**0.002**	0.244	0.427	0.714
Topographic pupil diameter (mm)	3.40 ± 0.70	3.41 ± 0.65	3.41 ± 0.78	3.41 ± 0.69	3.39 ± 0.61	0.704	0.759	0.693	0.440
AD (mm)	3.13 ± 0.65	3.13 ± 0.26	3.12 ± 0.27	3.12 ± 0.35	3.14 ± 0.26	0.581	0.537	0.505	0.309
ACV (mm^3^)	154.83 ± 28	154.53 ± 27	155.62 ± 26	155.44 ± 27	155.51 ± 27	0.821	0.554	0.618	0.620
ICA (angle)	42.42 ± 5.14	42.25 ± 5.41	42.31 ± 5.01	42.40 ± 5.28	42.59 ± 5.02	0.652	0.740	0.694	0.634
LLD (mm)	12.12 ± 0.73	12.40 ± 1.56	12.27 ± 0.71	12.31 ± 0.65	12.25 ± 0.62	0.131	0.172	0.193	0.254
CV (mm^3^)	59.20 ± 4.16	59.58 ± 3.77	59.45 ± 4.08	59.49 ± 3.92	59.25 ± 3.90	0.089	0,183	0,214	0,799

^∗^ *p* < 0.05 was considered statistically different and was indicated as bold. CCT: central corneal thickness; AD: aqueous depth; ACV: anterior chamber volume; İCA: iridocorneal angle; LLD: limbus-limbus distance; CV: corneal volume; *p*1: before application and at the 1st minute; *p*2: before application and at the 5th minute; *p*3: before application and at the 15th minute; *p*4: before application and at the 30th minute (*p* values when compared).

## Data Availability

All types of data used to support the findings of this study have not been made available because of institutional regulations.
